# Anal sphincter function in conservatively managed rectal intussusception at long‐term follow‐up: A prospective anal acoustic reflectometry study with comparison to healthy volunteer data

**DOI:** 10.1111/codi.70455

**Published:** 2026-04-30

**Authors:** Alexander O'Connor, Matthew Davenport, Niels Klarskov, Abhiram Sharma, Dipesh H. Vasant, John McLaughlin, Edward Kiff, Karen Telford

**Affiliations:** ^1^ Faculty of Biology, Medicine, and Health The University of Manchester Manchester UK; ^2^ Department of Colorectal Surgery Manchester University NHS Foundation Trust Manchester UK; ^3^ Department of Obstetrics and Gynecology Herlev University Hospital Herlev Denmark; ^4^ Faculty of Health and Medical Sciences University of Copenhagen Copenhagen Denmark; ^5^ Neurogastroenterology Unit, Gastroenterology, Wythenshawe Hospital Manchester University NHS Foundation Trust Manchester UK; ^6^ Salford Royal Hospital Northern Care Alliance NHS Foundation Trust Manchester UK

**Keywords:** anal acoustic reflectometry, faecal incontinence, rectal intussusception, rectal prolapse

## Abstract

**Aim:**

Rectal intussusception (RI), proposed to be a progressive condition, is associated with internal sphincter dysfunction. This study examines sphincter function using anal acoustic reflectometry (AAR) at long‐term follow‐up.

**Methods:**

A prospective study of conservatively managed patients attending a tertiary pelvic floor unit. Clinical, AAR and symptom severity data were analysed at baseline and follow‐up (>5 years). Patients were grouped into intra‐rectal (Oxford I–II) or intra‐anal RI (III–IV). Data from asymptomatic volunteers were used to estimate the effect of ageing on AAR parameters.

**Results:**

Twenty‐nine patients (27 female; median age: 66 years) were recruited, with follow‐up at a median of 66 (IQR: 64–67) months. Twelve were diagnosed with intra‐rectal RI and 17 with intra‐anal RI at baseline. No patient developed an external rectal prolapse.

There were no differences in quality of life, faecal incontinence or constipation symptoms in either group at follow‐up (*p* > 0.05).

A non‐significant decrease in opening pressure, a measure of internal sphincter function, was seen in both intra‐rectal (−4.0 cmH_2_O; *p* = 0.530) and intra‐anal RI (−3.7 cmH_2_O; *p* = 0.287). In healthy volunteers, opening pressure was associated with age (*r* = −0.402; *p* < 0.001) and a decline of 2.90 cmH_2_O (95% CI: −4.35 to −1.50) would be expected after 5 years.

Incremental squeeze opening pressure showed a non‐significant increase in intra‐rectal RI (8.4 cmH_2_O; *p* = 0.099) and no change in intra‐anal RI (−1.7 cmH_2_O; *p* = 0.532).

**Conclusion:**

There were no significant changes in patient‐reported symptoms or anal sphincter function in conservatively managed RI at follow‐up, challenging the concept of a progressive condition with a detrimental impact on sphincter function.

**What does this paper add to the literature?**

Rectal intussusception has been proposed as a progressive condition and is strongly associated with internal anal sphincter dysfunction. In this prospective study of conservatively managed RI, at 5‐years, there were no changes in anal sphincter function or patient reported symptoms challenging this assumption with implications for management.

## INTRODUCTION

Rectal Intussusception (RI) is often associated with symptoms of faecal incontinence (FI) and obstructed defecation syndrome (ODS) [1, 2], although it may represent a normal phenomenon in over 20% of asymptomatic volunteers [3, 4]. Whilst the natural history and pathophysiology of RI are not fully understood, an association between the grade of RI and dysfunction of the internal anal sphincter (IAS) has been demonstrated [5, 6, 7]. Higher Oxford grades of RI [1] are associated with reduced anal sphincter function and older patient age, supporting a hypothesis that RI may be the start of a process that eventually leads to external rectal prolapse [5, 7, 8]. Despite this hypothesis, the available retrospective data suggest progression to external prolapse is rare [5, 9, 10]. Others have proposed that due to an association with age in cross‐sectional series, RI may progress between the internal grades (I–IV) over approximately 4 years per grade [5, 8]. However, there have been no prospective longitudinal studies of RI to investigate its natural history, and this may have implications for its management.

In contrast to conventional fixed‐diameter anal manometry catheters, anal acoustic reflectometry (AAR) is a ‘catheter‐free’ dynamic test of anal sphincter function. It uses distension of the sphincter muscle to assess its length–tension relationship at different sarcomere lengths [11, 12], which can be used to describe muscle (dys)function [13, 14]. AAR has confirmed a clear association between IAS dysfunction and increasing Oxford grades of RI [7].

We hypothesised that if RI progresses over time, IAS function should also deteriorate. Therefore, this prospective longitudinal study aimed to explore whether anal sphincter function deteriorates at long‐term (>5 years) follow‐up in patients with RI who have received conservative (non‐surgical) interventions for symptoms of FI or ODS. Results were compared with AAR data from healthy asymptomatic volunteers to establish the expected decline in sphincter function due to ageing alone.

## METHODS

### Participants

Patients with RI reported in a cross‐sectional study of rectal prolapse undertaken at a tertiary pelvic floor centre [7] were approached after 5 years following initial recruitment. All patients were symptomatic adults at initial recruitment with functional anorectal disorders including FI and ODS. All had since received individualised non‐surgical interventions delivered by a continence therapist including pelvic floor physiotherapy, pharmacological interventions to adjust stool consistency or improve evacuation, containment products, dietary advice and appropriate toilet technique training.

Patients were diagnosed with RI at initial recruitment on fluoroscopic defaecating proctography interpreted by a consultant radiologist with an interest in pelvic floor disorders and graded according to the Oxford prolapse grading system [1]. Patients were stratified into one of two groups according to the prolapse grade at initial recruitment:

1. Intra‐rectal RI (low‐grade RI)—Oxford grades I (high rectal) and II (low rectal)
2. Intra‐anal RI (high‐grade RI)—Oxford grades III (high anal) and IV (low anal)

### Evaluation

At follow‐up, patients provided further written informed consent before a clinical history was taken and an examination was performed. Current symptom severity data were recorded including the presence and pattern of FI (urge, passive or mixed) and symptoms of urinary incontinence and faecal leakage. The presence of ODS symptoms was also recorded, which included the perceived difficulty in evacuation, a sensation of incomplete emptying, straining or regular digitation. Details of obstetric, gynaecological, anorectal and abdominal surgery recorded at initial recruitment were updated if required. Patients completed the same symptom severity patient reported outcome measures with differences between initial recruitment and follow‐up recorded. FI and constipation severity were assessed using the St Mark's Incontinence Score (SMIS) [15] and the Constipation Scoring System (CSS) [16], respectively. Quality of Life (QoL) was assessed with the Manchester Health Questionnaire (MHQ), which assesses QoL impact across nine domains [17]. It was not possible to repeat the defecating proctogram examinations at follow‐up due to the ethical concerns about unnecessary exposure to ionising radiation without clinical indication.

### Anal acoustic reflectometry

The technique of performing an AAR measurement has been described previously following a standardised reproducible technique [12, 18, 19]. Using a highly compliant collapsible polyurethane bag occupying a negligible cross‐sectional area (0.4 mm^2^) placed inside the anal canal, dynamic measurements of anal canal function can be observed during distension and relaxation. A digital signal processor (ED‐1932; Knowles Electronics, IL, USA) transmits wide‐band sound waves into the bag and reflected acoustic impulses are used to calculate the cross‐sectional area [20]. Intra‐bag pressure is then simultaneously recorded by a transducer (SX30D, Sensym sensor systems). A total of 10 cycles of inflation and deflation are performed at rest before five cycles of assessing voluntary squeeze where the patient is asked to squeeze whilst the bag is inflated.

Measurements are calculated from a graph of minimum cross‐sectional area versus pressure. Five resting measurements are calculated including the opening pressure (Op, cmH_2_O), which reflects the pressure the anal canal starts to open during distension. The closing pressure (Cp, cmH_2_O) is the pressure at which the anal canal closes down again following an episode of distension. Opening elastance (Oe, cmH_2_O/mm^2^) and closing elastance (Ce, cmH_2_O/mm^2^) reflect the resistance of the anal canal to distension, or the resistance to it closing, respectively. Hysteresis (Hys, %) reflects the amount of energy dissipated during one cycle of opening and closing of the anal canal. Two measurements are calculated during voluntary contraction: squeeze opening pressure (SqOp, cmH_2_O) and squeeze opening elastance (SqOe, cmH_2_O/mm^2^). The difference between SqOp and Op is defined as the incremental squeeze opening pressure (IncSqOp, cmH_2_O). The clinical significance of these has been discussed previously [21, 22].

### Asymptomatic healthy volunteers (assessment of the association between AAR parameters and age)

In order to explore the effect of ageing alone on AAR variables, measurements from 87 healthy asymptomatic continent female volunteers (median age: 60 [range: 24–89]) obtained from previous work in our institution between October 2008 and September 2011 were analysed [22]. The correlation between each AAR measurement and the age of the volunteer were explored with a linear regression analysis.

### Statistical analysis

Statistical analysis was performed with SPSS® for Mac® (Version 29.0, IBM®, NY, USA). Continuous data are reported as median (interquartile range [IQR]), unless otherwise stated. The Wilcoxon Rank‐sum test was used for comparisons between related samples of non‐parametric data. The Kruskal–Wallis and Mann–Whitney *U* tests were used for comparisons between independent samples of nonparametric data. Categorical variables are reported as *N* (percentage) and compared with a chi‐squared test. Statistical significance was considered at the *p* < 0.05 level.

## RESULTS

**FIGURE 1:**
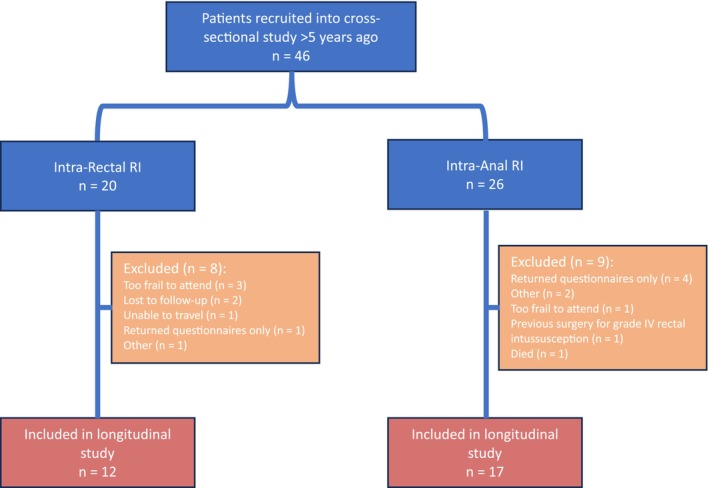
Patient recruitment and reasons for exclusion.

A total of 29 patients (27 [93%] female; median age: 66 years [range: 40–82]) were recruited into this longitudinal study from 46 eligible patients included in the original cohort. Seventeen excluded patients were unable to re‐attend our institution for further assessment (*n* = 13), were lost to follow‐up (*n* = 2), had died (*n* = 1) or had surgery for RI (*n* = 1) (Figure 1).

Of the included patients, 12 were diagnosed with intra‐rectal RI and 17 with intra‐anal RI at baseline. Follow‐up was at a median of 66 (64–67) months. Only one patient (1/46, 2%) had undergone surgery for grade IV RI and was therefore excluded.

Baseline and follow‐up demographic and clinical data are presented in Table 1. More patients in the intra‐rectal RI group had received neuromodulation therapy at follow‐up (*p* = 0.010). Table 2 illustrates patient reported symptoms at baseline and follow‐up. FI was the most common symptom across both groups. There were no statistically significant changes in symptomatology; however, more patients reported new ODS in the intra‐anal RI group at follow‐up (5 vs. 1; *p* = 0.168). None of the included patients reported developing an external rectal prolapse, and no external prolapse was identified on clinical examination at follow‐up.

**TABLE 1:** Baseline and follow‐up demographic and clinical data.

Parameter	Intra‐rectal RI (*n* = 12)	Intra‐anal RI (*n* = 17)	*p* value^a^
Age at baseline, median (range)	54 (34–71)	62 (48–77)	**0.016** ^b^
Age at follow‐up, median (range)	59 (40–76)	69 (54–82)	**0.018** ^b^
Male/Female, *n* (%)	1 (8%)/11 (92%)	1 (6%)/16 (94%)	0.798
**Baseline clinical data**			
Obstetric history, *n* (%)			
Parous^c^	10/11 (91%)	14/16 (88%)	0.782
Vaginal delivery^d^	9/10 (90%)	13/14 (93%)	0.803
Caesarean section^d^	6/10 (60%)	2/14 (14%)	**0.019**
Forceps use^e^	2/10 (20%)	5/14 (36%)	0.404
Ventouse use^e^	0	0	
Episiotomies or perineal tear^e^	9/10 (90%)	11/14 (79%)	0.459
Previous gynaecological surgery, *n* (%)^c^	7/11 (64%)	7/16 (44%)	0.310
Anterior repair	3	0	
Hysterectomy	1	2	
Sterilisation	1	2	
Other	2	3	
Previous anorectal surgery, *n* (%)	4 (33%)	4 (24%)	0.561
Rectocele repair	2	1	
Haemorrhoidectomy	0	1	
Other	2	2	
**Follow‐up clinical data**			
New gynaecological surgery, *n* (%)^c^	1/11 (10%)	2/16 (13%)	0.765
Hysterectomy	0	1	
Other	1	1	
New anorectal surgery, *n* (%)	2 (17%)	3 (18%)	0.945
Rectocele repair	1	1	
Other	1	2	
New neuromodulation treatment, *n* (%)	4 (33%)	0 (0%)	**0.010**
Percutaneous tibial nerve stimulation	3	0	
Sacral neuromodulation implant	2	0	

*Note*: Significance at *p* < 0.05 level. *p* values in bold are conisdered statistically significant.

Abbreviation: RI, rectal intussusception.

^a^ Chi‐squared test.

^b^ Mann–Whitney *U* test.

^c^ Females only.

^d^ Percentage calculated from parous females only.

^e^ Percentage calculated from females with vaginal deliveries only.

**TABLE 2:** Patient‐reported symptoms at baseline and follow‐up.

Symptom	Intra‐rectal RI (*n* = 12)	Intra‐anal RI (*n* = 17)	*p* value^a^
**Baseline symptoms**			
Bowel symptom history, *n* (%)			
ODS	6 (50%)	7 (41%)	0.638
Faecal incontinence	8 (67%)	15 (88%)	0.158
Urge faecal incontinence^b^	3/8 (38%)	1/15 (7%)	0.063
Passive faecal incontinence^b^	0/8 (0%)	5/15 (33%)	0.065
Mixed faecal incontinence^b^	5/8 (63%)	9/15 (60%)	0.907
Faecal leakage	3 (25%)	14 (82%)	**0.002**
Urinary incontinence, *n* (%)	5 (42%)	8 (47%)	0.774
**Follow‐up symptoms**			
Change in bowel symptoms, *n* (%)			
ODS			
New ODS	1 (8%)	5 (29%)	0.168
Resolved ODS	1 (8%)	0 (0%)	0.226
Faecal incontinence			
New faecal incontinence	0 (0%)	0 (0%)	n/a
Resolved faecal incontinence	1 (8%)	1 (6%)	0.798
Faecal leakage			
New faecal leakage	2 (17%)	0 (0%)	0.081
Resolved faecal leakage	2 (17%)	2 (12%)	0.706
New external rectal prolapse, *n* (%)	0 (0%)	0 (0%)	n/a

*Note*: *p* values in bold are conisdered statistically significant.

Abbreviations: ODS, obstructed defecation syndrome; RI, rectal intussusception; n/a, not applicable.

^a^ Chi‐squared test. Significance at *p* < 0.05 level.

^b^ Percentage calculated from patients with faecal incontinence.

There were no significant differences in patient reported outcome measures between baseline and follow‐up (Table 3). There was, however, a trend towards greater impact on quality of life in patients with intra‐anal RI at follow up (370.41 vs. 429.17), although this also did not reach statistical significance (*p* = 0.063).

**TABLE 3:** Patient‐reported symptom severity and quality of life measures at baseline and follow‐up.

Symptom severity measure	Intra‐rectal RI (*n* = 12)			Intra‐anal RI (*n* = 17)		
	Baseline	Follow‐up	*p* value^a^	Baseline	Follow‐up	*p* value^a^
Vaizey Incontinence Score	12 (2–20)	10 (6–15)	0.371	16 (11–18)	16 (9–18)	0.959
Constipation Scoring System	9 (1–12)	11 (6–13)	0.105	9 (4–11)	9 (7–14)	0.232
Manchester Health Questionnaire	469.59 (168.96–647.09)	447.09 (261.45–528.54)	0.814	370.41 (171.67–477.08)	429.17 (307.08–685.84)	0.063

Abbreviation: RI, rectal intussusception.

^a^ Wilcoxon Rank‐sum test. Significance at *p* < 0.05 level.

At follow‐up, most resting AAR parameters demonstrated a small overall reduction suggesting IAS dysfunction (Table 4). Opening pressure, a measure of IAS function, decreased in intra‐rectal RI (median difference −4.0 cmH_2_O) and intra‐anal RI (median difference −3.7 cmH_2_O); however, these differences were not statistically significant (*p* = 0.530, *p* = 0.287 respectively). With the exception of closing pressure in intra‐rectal RI (median difference +2.7 cmH_2_O), all other resting parameters reduced over the study period or remained unchanged, with no statistically significant differences observed. With squeeze parameters, incremental squeeze opening pressure (IncSqOp) increased in intra‐rectal RI (median difference 8.4 cmH_2_O) and showed no significant change in intra‐anal RI (median difference −1.7 cmH_2_O); however, these differences also did not reach statistical significance (*p* = 0.099, *p* = 0.532 respectively). Squeeze opening elastance demonstrated a small overall reduction without a statistically significant difference.

**TABLE 4:** Anal acoustic reflectometry results at baseline and at follow‐up.

Anal acoustic reflectometry parameter	Intra‐rectal RI (*n* = 12)				Intra‐anal RI (*n* = 17)			
	Baseline	Follow‐up	Median difference (IQR)	*p* value^a^	Baseline	Follow‐up	Median difference (IQR)	*p* value^a^
Opening pressure (Op), cmH_2_O	53.4 (39.0–65.1)	44.3 (33.2–74.7)	−4.0 (−15.6 to 7.5)	0.530	31.5 (24.7–47.6)	27.3 (19.7–38.0)	−3.7 (−6.8 to 5.2)	0.287
Opening elastance (Oe), cmH_2_O/mm^2^	1.8 (1.3–2.4)	1.6 (1.3–2.2)	−0.3 (−0.6 to 0.6)	0.695	1.1 (0.9–1.5)	1.1 (0.8–1.6)	−0.0 (−0.1 to 0.4)	0.943
Closing pressure (Cp), cmH_2_O	27.9 (20.2–44.4)	31.5 (20.7–51.1)	2.7 (−8.8 to 9.5)	0.638	19.7 (13.3–28.8)	15.9 (11.4–25.6)	−1.5 (−5.1 to 5.0)	0.831
Closing elastance (Ce), cmH_2_O/mm^2^	1.4 (1.2–2.1)	1.4 (1.0–2.0)	−0.2 (−0.4 to 0.2)	0.388	0.9 (0.8–1.3)	0.9 (0.6–1.4)	−0.0 (−0.2 to 0.4)	0.795
Incremental squeeze opening pressure (IncSqOp), cmH_2_O	22.0 (9.7–34.1)	30.5 (13.4–60.6)	8.4 (−4.6 to 24.3)	0.099	27.1 (7.5–87.6)	24.5 (16.6–66.0)	−1.7 (−18.0 to 10.2)	0.532
Squeeze opening elastance (SqOe), cmH_2_O/mm^2^	1.6 (1.3–1.9)	1.5 (1.1–1.8)	−0.2 (−0.4 to 0.2)	0.433	1.0 (0.9–1.8)	1.0 (0.8–1.8)	−0.1 (−0.3 to 0.0)	0.191

Abbreviations: RI, rectal intussusception; IQR interquartile range.

^a^ Wilcoxon Rank‐sum test. Significance at *p* < 0.05 level.

### The association of AAR parameters and age in healthy volunteers

Data from 87 healthy continent females (median age: [range: 24–89]) who were measured in our institution were examined to explore the effect of ageing alone on AAR parameters. Their association with age and expected decline after 5 years are presented in Supplementary Table 1. Age has a moderate negative association with Op (*r* = −0.402; *p* < 0.001) and Cp (*r* = −0.415; *p* < 0.001) and either a weak or no association with the remaining parameters. With Op, for each five‐year increase in age, Op would be expected to decrease by approximately 2.90 (−4.35 to −1.50) cmH_2_O.

## DISCUSSION

The grade of RI is known to be strongly associated with IAS dysfunction, with increasing RI grades associated with worsening resting anal sphincter tone [7, 23, 24]. This is the first prospective study aiming to explore the natural history of rectal intussusception based on this known association, using a novel test of anal sphincter function, anal acoustic reflectometry. It has identified that at long‐term follow‐up, there may be a small reduction in measurements of anal canal function at rest, with preserved squeeze function. These modest changes do not demonstrate statistical significance and would also appear unlikely to represent a clinically relevant deterioration. Whilst these changes may indicate worsening internal anal sphincter function associated with RI that may be progressing over time, when considered alongside the expected age‐related decline in AAR measurements in healthy volunteers, the results could be explained entirely by ageing. In addition, patients report stable symptoms, with the exception of an increase in ODS and a worsening quality of life impact in the intra‐anal RI group, whilst no patient developed external rectal prolapse over the study period. These findings challenge the assumption of a progressive condition, suggesting continued conservative management may be appropriate in some patients. Progression of RI may therefore only occur due to risk factors other than age or occur over a longer period of time than in this study.

AAR assesses the length–tension relationship of the sphincter muscle to measure its function [25, 26]. Previous work identified a significant association with deteriorating resting AAR parameters and an increasing Oxford grade of rectal prolapse [7]. In common with other authors using manometry, a strong association with IAS dysfunction and the grade of prolapse has been demonstrated. This has been proposed to be a consequence of direct trauma to the anal sphincter complex, impairment of the recto‐anal inhibitory reflex [27, 28], or may reflect a wider disorder of pelvic floor integrity leading to progressive weakening of support structures to allow the rectum to prolapse on straining [2, 23, 24, 29, 30, 31]. Alongside the association with deteriorating sphincter physiology, higher grades of RI have been reported in older populations in retrospective cross‐sectional series [5, 8]. However, in larger contemporary series, the reduced IAS function has been demonstrated to be independent of age [7, 24]. RI has been proposed to be a condition that progresses over time to higher grades of RI, or external prolapse [8]. However, increasing evidence now exists to suggest ageing alone may play only a limited role, with progression to external prolapse being a rare event (<4% over 5 years) [5, 9, 10]. Progression of RI may therefore occur only in some patients due to known or unknown risk factors including repeated and prolonged straining, a disorder of collagen synthesis, genetic predispositions [32, 33, 34] or a combination of several factors. It is also important to consider that in some instances ‘progression’ of RI observed on defecating proctography may be a consequence of the variability between repeat examinations influenced by both patient factors and interobserver variability with only a limited agreement between expert radiologists in the grading of RI in one large series (kappa, 0.29) of 105 examinations [5, 35, 36]. In our small series of symptomatic patients undergoing conservative management at a tertiary referral unit, the small decline in IAS function that may exist could be explained by ageing alone when examined against data from healthy volunteers. This suggests that whilst there may be progression of RI it is possibly a process occurring over several years beyond the follow‐up of this study.

The natural history of RI remains poorly understood, which has implications for its management. Notwithstanding the clear association demonstrated with IAS dysfunction and the Oxford grade, it remains unclear whether RI progresses, and if so, over what time period. There are also no clearly identified factors, which may influence its progression that could be modified. Alongside these uncertainties, RI can be identified on defecography in up to 20% of healthy asymptomatic volunteers [3, 4], and the examination itself is influenced by patient factors and inter‐observer variability, raising doubts about the correlation between this radiological diagnosis and patient reported symptoms. It is therefore no surprise that the most effective treatment for an individual patient remains unclear with unsatisfactory functional outcomes and high recurrence rates reported after surgery alongside concerns about potential serious adverse events [37, 38, 39, 40]. Indeed, in patients with constipation, work to coalesce review data highlighted that the evidence base is poor with inconsistent reporting of outcome measurements and a lack of long‐term data [41]. Whilst there were no significant improvements in patient‐reported symptoms in our study, where RI was managed conservatively, this finding could be helpful when discussing individual patient management options.

Further work is clearly required to understand the pathophysiology and natural history of RI to improve patient selection for intervention. Our study aimed to start to address this evidence uncertainty based on the known strong association with the grade of RI and IAS dysfunction, hypothesising that deteriorating IAS function may reflect progression of RI. The conclusions are limited by the small sample size and, due to ethical limitations, it was not possible to repeat the defecography examinations to demonstrate any radiological progression of RI should it exist, whilst its single centre design limits its generalisability. The data available from healthy volunteers may also be influenced by a proportion of these having asymptomatic RI, which may influence their measurements of sphincter function. Despite these limitations, this work should be considered as a call for further debate and research activity in RI within the colorectal community. It remains to be seen if the known IAS dysfunction is a reflection of a wider pathology affecting pelvic floor integrity that RI is simply a manifestation of, or if the prolapsing rectum leads to direct trauma to the sphincter complex. Assuming that the grade of RI and IAS dysfunction are correlated, the results in this study may suggest that conservative management could slow the progression of RI and the associated decline in sphincter function in some patients. Future work should now look at a larger cohort of patients specifically to address these uncertainties and address the question of progression of RI and its potential risk factors. Alongside this, work should begin to elucidate the pathophysiology of RI, which may have a basis in disorders of collagen synthesis or a genetic predisposition. It is hoped that this work could better inform patients and clinicians when selecting the most appropriate treatment strategy to ultimately improve functional outcomes.

## CONCLUSIONS

This prospective exploratory study has demonstrated that in symptomatic patients with RI there may be a small reduction in IAS function at long‐term follow‐up. When examined against data from healthy volunteers, this could be explained by ageing alone challenging the assumption of a progressive disease. Whilst it may represent a slow progression of RI over time, which may only become clinically evident beyond 5 years, further work is required to establish the natural history of RI in order to inform patient management.

## AUTHOR CONTRIBUTIONS

**Niels Klarskov:** Software; supervision; resources; writing – review and editing; validation. **Dipesh H. Vasant:** Conceptualization; methodology; validation; supervision; formal analysis; writing – review and editing. **Karen Telford:** Conceptualization; supervision; project administration; writing – review and editing; funding acquisition. **Matthew Davenport:** Conceptualization; formal analysis; data curation; writing – original draft. **Edward Kiff:** Conceptualization; data curation; investigation; validation; formal analysis; supervision; writing – review and editing; visualization. **John McLaughlin:** Supervision; project administration; writing – review and editing; methodology. **Abhiram Sharma:** Conceptualization; methodology; supervision; writing – review and editing; validation. **Alexander O'Connor:** Conceptualization; methodology; data curation; formal analysis; writing – original draft; funding acquisition.

## FUNDING INFORMATION

AOC received funding from The Royal College of Surgeons of England.

## CONFLICT OF INTEREST STATEMENT

The authors declare no conflicts of interest.

## ETHICAL APPROVAL

The data presented in this manuscript were obtained from two studies which both received ethical approval from the local research ethics committee (Greater Manchester—East [Ref: 17/NW/0547] and Greater Manchester—Central [Ref: 16/NW/0033]).

## CONSENT

All patients included in this study provided informed consent documented on a signed informed consent form.

## Supporting information


Table S1.

Data S1.

## Data Availability

The data that support the findings of this study are available on request from the corresponding author. The data are not publicly available due to privacy or ethical restrictions.
