# Supplementary material for: Anal sphincter function in conservatively managed rectal intussusception at long‐term follow‐up: A prospective anal acoustic reflectometry study with comparison to healthy volunteer data
Source: Colorectal Dis. 2026 Apr 30;28:e70455. doi: 10.1111/codi.70455 (PMC13130366; doi:10.1111/codi.70455)
Supplement: Supplementary file 1 — Table S1. [file CODI-28-0-s002.docx]

# Supplementary Tables

## Supplementary Table 1: The association with anal acoustic reflectometry parameters and age.

| AAR Parameter | r* | R^2^ | Linear Regression | 95% C.I. of slope | Expected Change in Parameter after 5 years (95% C.I.) | p value |
| --- | --- | --- | --- | --- | --- | --- |
| Opening Pressure (Op), *cmH_2_O* | **-0.402** | 0.161 | Op = 81.8 – 0.58(age) | -0.87 – -0.30 | **-2.90 (-4.35 – -1.50)** | **<0.001** |
| Opening Elastance (Oe), *cmH_2_O/mm^2^* | -0.068 | 0.005 | Oe = 1.3 – 0.002(age) | -0.009 – 0.005 | -0.01 (-0.05 – 0.03) | 0.530 |
| Closing Pressure (Cp), *cmH_2_O* | **-0.415** | 0.172 | Cp = 74.0 – 0.57(age) | -0.84 – -0.30 | **-2.85 (-4.20 – -1.50)** | **<0.001** |
| Closing Elastance (Ce), *cmH_2_O/mm^2^* | **-0.235** | 0.055 | Ce = 1.47 – 0.007(age) | -0.013 – -0.001 | **-0.04 (-0.07 − -0.01)** | **0.028** |
| Incremental Squeeze Opening Pressure (IncSqOp), *cmH_2_O* | -0.075 | 0.006 | IncSqOp = 51.5 – 0.18(age) | -0.69 – 0.34 | -0.90 (-3.45 – 1.70) | 0.503 |
| Squeeze Opening Elastance (SqOe), *cmH_2_O/mm^2^* | -0.158 | 0.025 | SqOe = 2.0 – 0.007(age) | -0.02 – 0.003 | -0.035 (-0.10 – 0.02) | 0.158 |
| *Pearson correlation coefficient with age. Significance at p < 0.05 level.  C.I. confidence interval | | | | | | |
